# The effects of prolonged running on foot posture: a repeated measures study of half marathon runners using the foot posture index and navicular height

**DOI:** 10.1186/1757-1146-6-20

**Published:** 2013-05-24

**Authors:** Emma Cowley, Jonathan Marsden

**Affiliations:** 1 School of Health Professions, Plymouth University, Faculty of Health, Education and Society, Peninsula Allied Health Centre, Derriford Road, Plymouth, PL6 8BH, UK

**Keywords:** Fatigue, Foot posture, Half marathon, Running, Pronation, Prolonged, Navicular height

## Abstract

**Background:**

Different foot postures are associated with alterations in foot function, kinetics and the subsequent occurrence of injury. Little is known about changes in foot posture following prolonged weightbearing exercise. This study aimed to identify changes in foot posture after running a half marathon.

**Methods:**

Foot posture was measured using the Foot Posture Index (FPI-6) and navicular height in thirty volunteer participants before and after running a half marathon. FPI-6 scores were converted to Rasch logit values and means compared for these and navicular height using an ANOVA.

**Results:**

There was a 5 mm drop in navicular height in both feet when measured after the half marathon (P < 0.05). The FPI-6 showed a side x time interaction with an increase in score indicating a more ‘pronated’ position in the left foot of + 2 [Rasch value + 1.7] but no change in the right foot (+ 0.4 [+ 0.76]) following the half marathon.

**Conclusion:**

The apparent differences between the FPI-6 and navicular height on the right foot may be because the FPI-6 takes soft tissue contour changes into consideration whilst the navicular height focuses on skeletal changes. The changes in foot posture towards a more pronated position may have implications for foot function, and therefore risk of injury; shoe fit and comfort and also the effect of therapeutic orthoses worn during prolonged running.

## Background

Pain and pathology affecting both bone and soft tissues due to mechanical overuse during prolonged running or walking have been frequently reported in the literature [1-11]. The foot has been reported to be the site of injury in long distance runners in 5.7- 39.3% of all reported running injuries compared to the ankle (3.9% to 16.6%), knee (7.2% to 50.0%) and lower leg (9.0% to 32.2%) [9].

A factor possibly linked to the incidence of lower limb injuries in athletes is foot posture. Relationships between occurrence of injury and foot posture have been shown in several studies [1,12,13] although with inconsistent findings. Burns et al. [13] investigated the effect of foot type on injury rates in 131 triathletes. They reported that supinated feet appeared to be related to higher rates of injury than pronated feet. This study, however, excluded foot orthosis wearers and, since it is mostly those with very pronated feet who wear foot orthoses, the authors acknowledge that they may have excluded a potentially important subgroup from the study. Burns et al.’s study builds on the findings of Cowan et al. [1] who investigated injury rates in 246 US Army infantry recruits and found that of the ‘high’, ‘normal’ and ‘low’ arched feet among the recruits that the low arched feet were at the least risk of injury with normal and high arched feet showing higher injury rates. Whilst these studies concurred that highly arched feet appear to be at greater risk of injury Williams et al. [14] surveyed twenty long distance runners with highly arched feet and 20 with low arches and found that both groups reported high levels of soft tissue injury and stress fractures. Further analysis of the data, however, revealed that the high arched runners tended to sustain more lateral foot and ankle injuries while those with low arches tended to sustain more medial foot and ankle injuries. These findings were consistent with their centre of pressure pathways which remained more medial in low arched individuals and more lateral in high arched individuals during a short, non-fatiguing run.

The effects of prolonged, more fatiguing exercise of the lower limb have also been investigated but still little is known about this affects the biomechanics and function of the foot and ankle [2,15-20]. The response to prolonged cyclic mechanical stress has been investigated in a repeated measures cohort study of 22 ultra-marathon runners [3]. This study found significant signs of strain and imminent pathology with soft tissue changes and bone marrow lesions being apparent during and after the race [3,20].

Since foot posture has been linked in some studies to varying function within the foot [21-23] any change in foot posture during prolonged running could also lead to a change in function and an associated risk of injury [1,24]. This study aims to assess the changes with foot posture immediately following a half marathon.

Measuring foot posture in fatigued athletes requires the use of validated, reliable and quick tools that could be performed simultaneously on several athletes. This precluded the use of a pedobarograph and a clinical test was deemed to be the most suitable. The foot posture index (FPI-6) and navicular height have been shown to have acceptable and good validity respectively [25] and are both quick tests to perform. Cornwall et al. [26] found that the FPI-6 had high intra-rater reliability but only moderate inter-rater reliability when used on both feet of 46 adult participants and similar findings were reported by Evans et al. [27] in 30 adult participants. Navicular height has also been tested for intra-rater and inter-rater reliability with better overall outcomes [27-29]. Evans et al. also found good inter-rater reliability for the truncated navicular height test and this concurs with findings by Williams and McClay [30] who found a moderate outcome with an intra-class coefficient (ICC) of 0.61 for this measurement between two testers in the navicular height test. Since the FPI-6 and navicular height were to be tested under timed and somewhat pressured conditions it was deemed necessary by the authors to establish the inter-rater reliability for these tests under the same conditions.

This study therefore aimed to measure the change in foot posture after running a half marathon using the FPI-6 tool and navicular height test. Such changes may have implications for shoe comfort and altered foot function at different stages of a long distance run.

## Methods

Ethical approval was sought and granted by Plymouth University, Faculty of Health ethics committee.

### Sample size calculations

Previous work on 3968 army recruits found that the navicular height was 40.4 mm ± 7.2 for people defined as having a normal plantar shape [31]. We estimated that to detect a 10% change in navicular height following the half marathon (an effect size of 0.4/7.2 = 0.56) would require 30 participants (power = 0.85; α = 0.05).

### Recruitment of participants

The study was advertised by the Plymouth Half Marathon website and Plymouth University website and the advert highlighted the need for healthy volunteers registered to run the Plymouth Half Marathon and who had no foot pain or pathology. Exclusion criteria was any participant with a history of significant foot injury or surgery, current foot pain or on-going use of foot orthoses, a diagnosis of diabetes mellitus or arthritis affecting foot and ankle joints.

No volunteers were excluded upon initial assessment and the first 30 gave informed consent (12 female and 18 male, aged 20–53 yrs (median 35 yrs), BMI range 16.6-29.7 (median 24.6)). Footwear was not controlled for in this study although no runners trained or raced in footwear deemed by the primary author to be unsuitable for their foot type. Most runners wore ‘stability’ type running shoes. All footwear worn by the participants was in good condition and runners were advised to tie shoe laces well in order to minimise foot slippage in the shoe and ensure a good fit throughout the race.

### Data collection

The second tester (MM) was an inexperienced podiatrist and so significant time and training was given by the primary author (EC) to ensuring competence in using both the FPI-6 tool and measuring navicular height. Inter-rater reliability testing was undertaken on 10 healthy participants who were measured by each rater 10 minutes apart. The ICC was calculated in SPSS (version 20 IBM). The ICC for the FPI-6 was 0.99 and 0.98 for navicular height indicating excellent reliability.

In the week prior to the half marathon participants attended a pre-race measurement session in a non-fatigued state having been instructed not to run on the day. The primary author (EC) who was a podiatrist of fourteen years’ experience collected all the pre-race data.

Height, weight and age were recorded and then participants stood in relaxed stance with each foot on an analogue weighing scale and a 10 cm inter-malleolar gap. With weight evenly distributed across each foot the Foot Posture Index (FPI-6) score was recorded. Similarly navicular height at the most medially prominent point of the navicular tuberosity was marked on stiff card using the technique described by described by Brody [32] and records were kept using the runners’ race numbers to identify participants. The participants were given instructions for race day to immediately attend the research station for post-race measurement, using the same measures, in their fatigued state.

Both EC and MM undertook post-race data collection due to the runners arriving at the research station in quick succession although EC was the default first choice where both data collectors were available and she collected the majority of data. By ensuring the next available data collector recorded the measurements the time to measurement for the runners remained under five minutes from the race finish time and ensured the runners were measured in their fatigued state. All runners arrived at the station, were seated and asked to remove their footwear by helpers in the research team and briefly questioned and examined for injuries sustained during the race. Minor injuries such as open erosion lesions, blisters and toe nail lysis were not considered likely to affect FPI-6 or navicular height unless they directly affected the areas needed to record the data. No runners sustained injuries in these areas. Furthermore, more serious injuries such as ankle sprains, or significant musculoskeletal pain would have affected the measurements and participants would have been excluded from the study in this instance. No runners were found to have sustained such injuries and all thirty remained in the study.

It was an ethical consideration that runners would be considerably fatigued after the race and would possibly require immediate rest and refreshment prior to measurement. This was allowed for during the initial seating and shoe removal stage where members of the research team aided them in shoe removal if necessary and ensured they were able to stand in order to be measured. One runner felt faint upon arrival at the station and was allowed time to eat a snack in order for him to be able to stand safely on the research stand and another suffered calf cramps for a couple of minutes prior to data collection but we were still able to obtain measurements within the five minutes following their race finish times. To ensure that weight was evenly distributed between left and right feet and that upright posture was maintained during measurement the runners placed each foot on an analogue weighing scale, as in the pre-race measurement, and data were only recorded when each foot was taking 50% of bodyweight. The stand also included a waist height hand rail in front of the runner to enable them to maintain balance.

### Statistical analysis

The FPI-6 is an ordinal tool and as such is not suited to parametric statistical testing. The original Foot Posture Index in both its original 8 factor form and latter 6 factor form was analysed using the Rasch model by Keenan et al. [33] and as part of the process each FPI-6 factor measurement was attributed a ‘logit’ value to represent the tool’s ordinal data points. The logit values described by Keenan et al. [33] enable this study’s FPI-6 data to be analysed using parametric tests using the sum logit scores for each participant. The Rasch scores can be interpreted in the same way as the ordinal scores as they still directly relate to a positive and negative (pronated – supinated) scale as the original FPI-6 scale.

Navicular height and Rasch logit converted foot posture index scores were normally distributed as determined by the Kolmogorov-Smirnov test (P > 0.05) and parametric tests were therefore selected. Results were analysed in SPSS (version 20 IBM) using a repeated measures analysis of variance with factors being TIME (pre vs post-race) and SIDE (right vs left). A stepwise multiple regression investigated the relationship between the change in foot posture as measured using the FPI-6 or navicular height and the persons’ age, gender, BMI, race time and pre-race foot posture. Results were taken as significant if p < 0.05; mean and standard deviations are reported unless indicated.

## Results

Thirty people were assessed (12 female and 18 male, aged 20–53 yrs (median 35 yrs), with a BMI range of 16.6-29.7 (median 24.6)). The average time to complete the race was 124.6 ± 2.4 minutes. Baseline median FPI-6 was + 3 (ordinal score) for both feet (Inter Quartile range = 3.5) and median navicular height was 47 mm (mean = 47.39 mm). The pre and post-race results are shown in Table 1.

**Table 1:** Pre and post-race mean FPI-6 (Rasch logit value) and Navicular height measures are indicated (± standard deviation)

**Parameter**	**Pre-race**		**Post-race**	
	**Left**	**Right**	**Left**	**Right**
Navicular Height (mm)	46.4±6.5	48.4±8.0	42.2±6.6	43.2±4.8
FPI-6	1.8±1.5	1.3±2.2	3.4±2.3	2.1±2.2

### Navicular height

**Figure 1:**
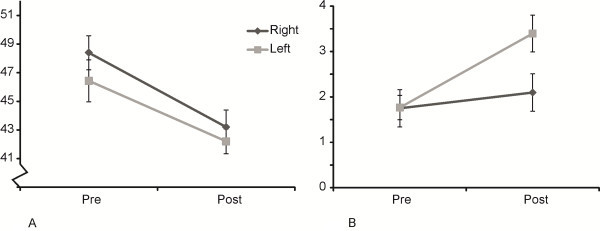
**Change in navicular height (A) and FPI-6 (B) with a half marathon.** Decreases in navicular height and increases in positive FPI-6 values indicate an increase in pronated foot position. Mean ±SEM is indicated.

Mean navicular height significantly decreased following the half marathon by 5 mm (TIME F (1,29) = 26.9 p < 0.001, Figure 1A). There was no effect of side or side x time interaction.

### Foot posture index

The FPI-6 scores [Rasch logit values] significantly increased following the half marathon (TIME F (1,29) = 15.9 p < 0.001). There was a significant time x side interaction (TIME X SIDE F (1,29) = 15.1 p < 0.001). The interaction indicates that following the half marathon the FPI-6 scores increased significantly more on the left [+ 1.7] and although the right side changed too [+ 0.3] this was not statistically significant (Figure 1B).

### Relationship between participant characteristics and change in foot posture

There was no relationship between the change in FPI-6 scores or navicular height with the half marathon and the participants’ age, gender, BMI, race time or pre-race foot posture as assessed by the average FPI-6. Where the mean pre-race navicular height (pre_n) was higher there was a significantly greater drop in navicular height post-race (ch_n) (R^2^ = 0.39, F(1,27) = 17.05 P < 0.001; ch_n = −0.45 x pre_n +17).

## Discussion

This study showed that arch height tended to decrease after running a half marathon although there were differences between the two measures used. Age, gender, BMI, race time did not predict the change in arch height. The change in arch height was larger in people with a higher pre-race navicular height.

A significant drop was seen in the left FPI only. This may reflect the influence of leg dominance resulting in differences in lower limb kinematics and kinetics between the two sides. However, this is speculative and leg dominance was not recorded in the current study. A more likely explanation is that this reflects the limited sample size; to detect an effect size of 0.38 on the right would require a sample size of 137 participants (power = 0.85 α = 0.05).

The definition of foot pronation by Root et al. [34] describes the arch lowering through subtalar joint pronation as one of the observable clinical signs. More recently Nester [35] reviewed experimental evidence which challenged Root’s explanation of the arch lowering primarily through subtalar joint pronation. Nester indicated that arch lowering through sagittal plane plantarflexion and frontal plane eversion is found variably between individuals to be comprised of movement primarily at the ankle and talonavicular joint and to a lesser extent through the other tarsal joints. It is difficult, therefore, to infer which soft tissue structures may be yielding in any one individual to lower the arch under weightbearing conditions following a prolonged run. It is possible that creep of passive soft tissues upholding the medial longitudinal arch may have occurred and also muscle fatigue in anti-pronatory muscles. The arch profile frequently changes upon weightbearing and so the navicular moves as a result of whichever combination of joint movements has occurred, and can be used as a measure of the weightbearing response of the foot which is known clinically as pronation and supination [36].

Another effect of exercise, however, is the perfusion of blood to the muscles of the foot which results in engorgement and an increase in volume. The muscles closest to the skin in the medial longitudinal arch contribute to the maintenance of arch height [37] and so would likely be placed under considerable work during a half marathon with significant engorgement occurring during a race. Since the increase in volume would fill the medial longitudinal arch space this would give the impression of lowering of the arch irrespective of navicular height. Indeed some feet appeared to have increased in volume at the medial midfoot perhaps resulting from increased perfusion to the abductor hallucis and other local muscles. Engorgement and an increase in blood volume could potentially affect the interpretation of the FPI, which is done visually, whilst the measure of navicular height to a bony landmark should be unaffected.

Anecdotally the testers noticed an apparent abduction of the forefoot relative to the rearfoot post-race which may result from fatigue of the medial foot soft tissues which are the stabilisers of the tarsus in the transverse plane. This could even be a distortion of the shape of the foot due to superficial muscular engorgement both medially and laterally. It would be useful to investigate the transverse plane response of the foot to prolonged running and walking in future studies.

The navicular height pre-race was ~ 5 mm higher than that recorded by Swedler et al. [31] in large scale study of army recruits. This may reflect the difference in the points of measurement; the inferior border of the navicular bone in the study by Swedler et al. [31] and the navicular tuberosity in the current study. One of the drawbacks with this study, however, is that we did not normalise navicular height to the length of the foot. People with larger feet would tend to have a higher navicular height and show a larger change in height after running. This may explain why a significant relationship was seen between the change in navicular height and the pre-race navicular height. However, the FPI-6 also showed an increase in pronated position and this tool does not normalise for foot length. The fact that the degree of navicular drop was not related to the pre-race FPI score whilst this was related to pre-race absolute navicular height suggests that the degree of arch lowering is the same regardless of foot length.

Time to return to baseline FPI-6 score and navicular height was not recorded for the participants and further research into this might be useful. The cause of the changes in foot posture seen in this study are not clear and could be the result of damage to soft tissues or yielding within elastic limits in the soft tissues due to neuromuscular and mechanical fatigue. The impact of these changes on foot and ankle function is also unknown nor the effect of running a longer distance than a 13.1 mile half marathon. The changes may indeed be clinically significant enough to predispose the bones, joints and soft tissues to damages if running were to continue after the changes have taken place, for example in a full marathon. The levels of pain anecdotally reported after the race by the runners were not considered to indicate significant injury but this may be erroneous due to general systemic fatigue and raised endorphin levels which have been reported in trained athletes during sporting activity [38] The perception of pain and function may be potentially important in modification of activity after clinically significant changes have occurred and should be investigated further.

We did not control for footwear in this study although discussion about footwear was offered in the pre-race data collection sessions and advertised as an incentivisation for the study. Participants were advised to continue with their planned footwear for the race and consider any advice given by the researcher EC when only buying new training footwear in future. Participants were advised on lacing techniques, however for the race to ensure that shoes fitted well to reduce the risk of skin erosions.

Biomechanical studies of low and high arched runners have, demonstrated an effect of cushioning and motion control sports shoes on the biomechanics of the lower limb during treadmill running [39]. They found that low arched runners have more internal tibial rotation when wearing cushion shoes and less when wearing motion control shoes whilst no effect on internal tibial rotation was seen in high arched runners. As internal rotation is associated with foot pronation, this suggests that the type of shoe may be a factor in determining changes in foot posture with running.

## Conclusions

This study showed a change in foot posture to a more pronated position following running a half marathon race. The magnitude of increase in pronated foot position in different foot types, changes in function and time to recover original foot posture and the impact of footwear are not yet known. Clinicians managing running injuries relating to pronated foot position may find the results of this study helpful when considering therapies in long distance runners.

## Competing interest

The authors declare that they have no competing interests.

## Authors’ contribution

EC was responsible for study design, data collection and analysis and dissemination. JM was responsible for study design, ethics application, analysis and dissemination. Both authors read and approved the final manuscript.
